# Community’s knowledge, perceptions and preventive practices on Onchocerciasis in Jimma zone, Ethiopia, formative mixed study

**DOI:** 10.1371/journal.pntd.0011995

**Published:** 2024-03-13

**Authors:** Daba Abdissa, Yohannes Kebede, Morankar Sudhakar, Gelila Abraham, Gebeyehu Bulcha, Teshome Shiferaw, Nimona Berhanu, Firanbon Teshome, Hirpa Miecha, Zewdie Birhanu

**Affiliations:** 1 Department of Biomedical Sciences, Jimma University, Jimma, Ethiopia; 2 Department of Health, Behavior and Society, Jimma University, Jimma, Ethiopia; 3 Department of Health Policy and Management, Jimma University, Jimma, Ethiopia; 4 Jimma Zone Health office, Oromia, Ethiopia; 5 School of Pharmacy, Jimma University, Jimma, Ethiopia; 6 Oromia, regional health bureau, Oromia, Ethiopia; Federal University of Agriculture Abeokuta, NIGERIA

## Abstract

**Background:**

In Ethiopia, Onchocerciasis is a prevalent neglected tropical disease, currently targeted for elimination with mass drug administration and community behavioral changes towards sustained control and eventual elimination. This study aimed to elucidate the awareness, perceptions and practices of endemic communities in Jimma Zone, Ethiopia.

**Methods and materials:**

Community-based cross-sectional study triangulated with qualitative method was conducted from October-November, 2021. A multistage sampling was employed and data were collected using a pre-tested interviewer-administered structured questionnaire. Logistic regression was used to identify the predictors of comprehensive knowledge and preventive practice. Adjusted odds ratios were calculated at 95% confidence interval (CI) and considered significant with a p-value of <0.05. Kruskal-Whallis and Mann-whitney tests were used to compare median risk perception score by socio-demographic factors. Qualitative data were collected through focus group discussions and key informant interviews and transcribed verbatim. Then the data were coded, categorized, and themes were developed.

**Result:**

The overall prevalence of adequate comprehensive knowledge was 48.8% (95% CI: 44.9, 52.3), high risk perception was 18.7% (95%CI15.9, 21.4) and preventive practice was 46.9%(95%CI:(43.3,50.4). High risk perception[AOR = 1.95 95%CI: (1.32, 2.89] was statistically significant with comprehensive knowledge, likewise knowledge of mode of transmission [AOR = 2.64 95% CI: (1.44, 4.85)], knowledge of consequences [AOR = 2.12 95%CI: (1.21, 3.72)] and knowledge of preventive measures [AOR = 15.65,95%CI:(10.1, 24.2)] were statistically significant with preventive practice. The median risk perception was varied significantly between the groups by educational status, study district and age category. Qualitative evidence showed that there were great community knowledge gap about the disease.

**Conclusion:**

Community knowledge, perceptions, and practices are unacceptably low. Risk perception was significantly associated with comprehensive knowledge, likewise knowledge of mode of transmission, consequences and preventive measures were significantly associated with preventive practice. This implies knowledge is a key component of effective prevention strategies as it is a necessary condition for the behavior change.

## Author summary

Onchocerciasis is a common neglected tropical disease with serious consequences in Ethiopia. Mass drug administration and behavioral changes in the community are being used for its control and eventual eradication, but community awareness, perceptions, and practices are still underreported. This study examined community knowledge, perceptions and preventive practices towards Onchocerciasis using mixed method in Jimma, Ethiopia. The finding revealed that, most of the respondents were familiar with Onchocerciasis, despite some confusions and misinterpretations around it. In these communities where Onchocerciasis is endemic, correct perceptions and proper understanding of this disease was inadequate and the level of risk perception towards Onchocerciasis was quite low. On the other hand, more than half of the participants did not practice Onchocerciasis recommended preventive measures and lack adequate knowledge of it. Furthermore, participants who were knowledgeable about the mode of transmission, its consequences and preventive measures were more likely to practice its preventive measures. Thus, our findings highlight the need for strengthening context-specific and tailored behavior change communications by integrating it into routine Onchocerciasis control and elimination program.

## Background

Onchocerciasis which is caused by the parasite *onchocerca volvulus*, is one of the neglected tropical diseases (NTDs) that frequently cause devastating illness in low-income countries [1]. It is transmitted by the repeated bites of infected Simulium blackflies that breed in fast-flowing rivers [2]. It primarily affects the working-age population in rural Africa’s tropics and subtropics [3]. It was the world’s second-leading infectious cause of blindness in 2015[4] and it is a stigmatizing disease that causes severe psychological stress [5].

According to the Global Burden of Disease Study, there were 20.9 million Onchocerciasis infections in 2017[6]. About 125 million individuals globally were at risk of Onchocerciasis with 96% of those people living in Africa [7] and more than 99% of cases taking place in rural parts of sub-Saharan Africa living near rivers [8]. Onchocerciasis is highly endemic in Ethiopia. As the world health organization (WHO) reported in 2016 about 20 million people live in the endemic areas and 3 million people are infected with it [9]. It is endemic in 188(24.4%) districts of Ethiopia [10,11]. According to the 2016 report by the Ethiopian Federal Ministry of Health, 5.8 million people live in highly endemic areas [11]. Onchocerciasis is reported to be endemic in Ethiopia’s western Oromia Region, Southern Nations Nationalities and Peoples’ Region, northwestern Amhara Region, larger part of Gambella and Benishangul-Gumuz region [12,13]. The prevalence of Onchocerciasis in Ethiopia ranges from 6.9% in the Quara district of Northwest Ethiopia to 74.8% in West Wollega, Ethiopia [14,15]. Additionally, a 2015 study from Jimma found that its prevalence was 22.5% [16].

The primary risk factors that put people at risk for being bitten by Simulium blackflies (onchocerca volvulus vector) are farming, living or working close to streams or rivers where Simulium blackflies are present, washing clothes next to streams or rivers, and swimming in streams or rivers [17]. The blackflies breed in running water, though some species live in large, fast-flowing streams, and others in small, sluggish rivulets. These blackflies exclusively prefer clean and healthy streams because it does not tolerate organic pollution. Female blackflies lay their eggs on vegetation, in streams or scattered over the water surface where they grow into adult blackflies that search for food (blood meal) so they can lay eggs to continue the life cycle. In relation to the habitant nature of blackflies, Onchocerciasis has been a significant public health issue, particularly in large-scale coffee plantation areas of Southwest Ethiopia that are densely populated, enormously covered in forests, and have abundant perennial rivers and streams [18]. Thus, people who are closely engaged in rural agricultural farming activities near running rivers/streams are at highest risk for acquiring Onchocerciasis, since they are repeatedly bitten by blackflies. During a blood meal, female blackflies inject infectious L3-stage larvae into a human. Thus, people who are close engagement in rural agricultural farming activities near running river/stream are at most at risk for acquiring Onchocerciasis since they get repeated bite from the black flies. During a blood meal, female blackflies inject infectious L3-stage larvae into a human. The worms mature in the human host after one to three years, and adult worms (macrofilariae) live in nodules beneath the skin, where the female worm can survive for up to 15 years. The adult female worm releases hundreds of motile microfilariae each day, which migrate beneath the dermis of the skin and cause extremely inflammatory reactions [19,20].

Onchocerciasis is responsible for substantial morbidity, psychosocial problems, reduced capacity for work, and decreased agricultural productivity. Serious socioeconomic effects, intense itchiness and blindness are caused by it [21,22]. The social effects of Onchocerciasis are severe and include declines in economic production as well as severe social stigmatization, especially among women, which has an adverse effect on the sexual health of those who are infected as well as on social interactions and self-confidence [23–25]. The disease has several long and short term consequences including but not limited to reduce life expectancy, poor sleep, poor academic performance and a greater dropout rate in infected school-aged children epilepsy [26–28] as well as significant medical costs, lowers agricultural production and perpetuation of poverty [29].

In accordance with WHO strategy, the Ethiopian government launched a control and eradication program for Onchocerciasis and other NTDs. Mass drug administration (MDA) using Ivermectin has been one of the promising strategies for interrupting Onchocerciasis transmission and for the gradual elimination of the disease, contributing to the national and global effort to eradicate NTDs by 2030[30]. Ethiopia started the MDA program in 2001 through community drug distributors, and continued until 2012 through annual Ivermectin MDA in all meso and hyper- endemic areas of the country. In 2012, the program goal changed from control to elimination with a shift to a biannual MDA approach [31,32].

The infection is still prevalent in most foci in Africa with prevalence rates of up to 50%, despite the use of Ivermectin MDA for several years [16,33]. Although the reported coverage of MDA is encouraging, Onchocerciasis has continued to spread in many parts of Ethiopia [32]. Even though the Federal Ministry of Health of Ethiopia set a goal to eliminate Onchocerciasis and to stop community-directed treatment with Ivermectin by the end of 2020, the deadline was shifted to 2025 due to the potential transmission of the disease in the endemic areas [13]. To control and eradicate this illness, complete geographic coverage and consistently high Ivermectin coverage for at least 15 to 17 years of high coverage (80%) are required [34,35]. In addition to mass distribution of Ivermectin in endemic areas, Ethiopia also adopted complementary control and prevention strategies such as vector control using personal protection measures against biting insects, treatment of cases and other awareness raising activities to prevent bites [36].

Despite these efforts, however, treatment coverage was suboptimal in many endemic settings of Ethiopia [31,37,38] due to many factors such as low-risk perception and misperceptions about the disease [39,40], absenteeism at the time of the campaign, experience of side effects in the past [39], knowledge of the disease, level of education received on MDA [41], suboptimal program implementation, distrust of the drug distributors [40,42], low knowledge of the benefit of the drug [39,42,43], perceived social influence and support [44], COVID-19 pandemic, logistical challenge and civil unrest [42,45].

One of the highest priority intervention areas in the context of elimination of Onchocerciasis is raising and maintaining high levels of knowledge and appropriate preventive practices among populations living in endemic areas in order to achieve long-term onchocerciasis control, elimination goals, and to strengthen locally acceptable and suitable control measures [32,46,47]. This is because Onchocerciasis elimination initiatives require a higher level of active community engagement [32,48]. The efficacy and effectiveness of Onchocerciasis control measures depends on the community’s accurate understanding of the disease as well as its perception and preventive actions [32,47].

Maintaining community awareness and increasing appropriate community responses become more difficult, however, as the disease’s incidence declines. Communities start to perceive they are no longer at risk for Onchocerciasis, which contributes to continuity of disease transmission. Thus, it is imperative to evaluate a local community’s awareness, perceptions and practices towards Onchocerciasis and its prevention measures in areas where an elimination effort such as MDA is underway. Though Onchocerciasis is endemic in southwest Ethiopia [16], there is inadequate information regarding the community’s level of knowledge, perceptions and practices with respect to the control and preventions of Onchocerciasis. Moreover, there is little up-to-date information especially in the context of elimination targets, despite the fact that a few studies which attempted to assess community’s knowledge of Onchocerciasis and preventive practices in some parts of Ethiopia [2,16,49,50].

The purpose of this study, therefore, was to assess community knowledge, risk perception, preventive practices and related factors of Onchocerciasis as well as a triangulation of the qualitative study findings for a comprehensive understanding of the phenomenon in Jimma zone, Ethiopia. The results would be useful to develop context-specific community education and awareness building interventions to aid ongoing Onchocerciasis prevention, control, and elimination efforts in Ethiopia and other similar areas.

## Methods and materials

### Ethical statement

The study protocol was received and approved by an ethics review committee of the Institute of Health, Jimma University (ref No: JHRPGD/344/2021). The purpose of the study was explained to each respondent and each participant was received detailed information about the study, benefits, and risks of participation and participation process. In order to participate in the study, all adults (age ≥18) were signed a written consent form. For participants under the age of 18, written parental consent was taken from the parent (guardian) in accordance with the Declaration of Helsinki. The privacy and confidentiality of the information were ensured. All COVID-19 preventive measures were applied during data collection.

### Study area and design

A community-based cross-sectional study supported by qualitative study (**S1 Material)** was conducted in five Onchocerciasis-endemic districts (Gomma, Manna, Kersa, Omo Neda (O/Neda), and Omo Beyam (O/Beyam)) of Jimma zone, from October-November, 2021. Jimma zone is located 357 km to the west of Addis Ababa, the capital of Ethiopia. Based on the 2007 Census conducted by the Central Statistics Agency, this zone has a total population of 2,486,155 and 22 districts [51]. The chosen district had the following total populations: Gomma (213,023), O/Nada (248,173), Kersa (165, 391), O/beyam (145, 573), and Manna (146,675) [52,53].

Jimma Zone is situated at approximately 7.6599° N latitude and 36.8327° E longitude, Jimma Zone boasts rolling hills, fertile plains, and an ideal climate for agriculture, all within a vibrant cultural and economic hub celebrated for its warm and welcoming people. Manna district, located around 7.5100° N, 36.8100° E, is famed for its flourishing fields and vibrant communities, while Gomma district, at about 7.3556° N, 36.8654° E, imparts a unique character to the region with its distinctive features. Positioned approximately at 7.8000° N, 36.9000° E, Kersa district holds significance for its rich cultural heritage and natural beauty. Nestled at around 7.6000° N, 36.9500° E, O/Nada district offers valuable insights into the diverse districts within the zone, and located at about 7.7167° N, 36.7500° E, O/Beyam district forms an integral part of the Jimma Zone’s diverse and dynamic landscape [54].

The study area is marked by the existence of a robust healthcare system, encompassing two main hospitals and 36 health centres, underscoring the presence of a substantial healthcare infrastructure. It is well-known for its coffee production, which is a key economic activity in the region. Additionally, it boasts an abundance of major rivers that play a pivotal role as breeding grounds for black flies, such as the Aleltu and Dogoso rivers in Kersa, the Dige river, the Kolombo river, and the Naso river in Gomma, the Gube Bosoka river in Manna, and the Beyam river in O/Nada, including O/Beyam. Notably, the majority of these rivers are utilized for agricultural irrigation and domestic purposes [53]. Given these factors, this study offers an excellent opportunity to better understand the complex interplay between disease transmission and local ecological and economic factors in the zone [55].

### Study population

All selected spouses of heads of household (women) who had resided in the district for at least six months comprised our study population. For the qualitative part of the study, a diversified population including community members, school students and frontline health workers were participated in the study.

### Eligibility criteria

All spouses of heads of household who are residents in the district for at least six months were included, where as those who were unable to communicate and residents of less than six month were excluded from the study.

### Sample size determination and sampling technique

This study was part of larger baseline study aimed to evaluate the effectiveness, feasibility and acceptability of co-delivery of two MDA for Onchocerciasis and soil transmitted helminthes. The sample size for the baseline and coverage survey was calculated using a single proportion formula, *n* = [DEFF*Np (1-p)]/ [(d^2^/Z^2^_1-α/2_*(N-1)+p*(1-p)] assuming 75% effective of campaign coverage for Soil transmitted helminthes (reach) [56] of the eligible population who participated in the campaign; design effect of 1.5, and marginal/confidence limits as 4%, and 10% non-response rate, which yields 743 sample size. The final sample size was large enough to form a representative sample.

A multistage sampling was employed to select the participants and the sampling procedure was performed as follows: First, five districts were chosen from a total of 22 in the Jimma zone. The selection of the study districts was based on input from local health experts and consideration of criteria such as disease endemicity and the focus of MDA efforts. From each selected district, two Gandas (lowest administrative unit in Oromia, Ethiopia, equivalent to village) were selected randomly for inclusion into the study; making a total of ten villages. The complete sample was then proportionally distributed to designated districts. Similarly, the sample sizes were assigned proportionally to randomly selected villages in each of the five selected districts. Finally, simple random sampling technique was employed to select the study participants for inclusions in to the study. Our sampling unit was household and the study unit was spouses of head of household. The data were collected primarily from women (spouses) in the households. In cases where a woman was not present, an adult male (spouse) was considered, and if neither spouse was there, an adult family member who could provide information was interviewed. For the sake of sampling frame and aim of the main project, census was conducted.

For qualitative part, the sample size was estimated based on the ultimate objective of the qualitative evidence required and the saturation of ideas was taken into consideration to stop further sampling. Accordingly, 4 key informant interviews (KIIs) with community volunteers and community leaders were conducted **(S2 Material)**. Besides, 6 focus group discussions (FGD) with male and women community members, and school boys and girls were carried out. The number of participants of each FGD ranges from 7 up to 11. One expert group discussion with rural health extension workers with more than 8 years of experience was also conducted. Recruitment of the participants was conducted considering different factors such as area, experiences and position.

### Data collection tool and procedure

A structured interviewer-administered tool that was developed from pertinent literatures [2,8,11,16,49,50] and adapted to the local context to ensure relevance and content validity was utilized. The questionnaire was then translated into Afan Oromo (local language), pre-tested in a similar area, and collected by trained data collectors with first degree holders. The questionnaire consisted of different sections including socio-demographic characteristics, knowledge, risk perception and preventive practices related to Onchocerciasis. Knowledge, perception, and preventive practices were the study’s outcome variables, which were measured and defined as follows.

### Measurements and scoring procedures

#### Knowledge on Onchocerciasis

Multi-dimensional knowledge (MK) about Onchocerciasis (consequences, symptoms, mode of transmissions and prevention) was measured using yes/no questions. Accordingly, respondents’ knowledge of basic sign and symptoms of Onchocerciasis was assessed using five classical symptoms of Onchocerciasis (i.e. intensive itching, skin rash, eye itching, firm nodule in the skin and skin color change); knowledge of mode of transmissions using 2 items (i.e. blackfly bite as transmitting factor of Onchocerciasis and swimming/washing with stream water/canal water risk factor), knowledge of consequences of Onchocerciasis using 3 items(i.e. blindness, skin scar/discolorations/disfigure and social stigma); and knowledge of preventive measures using four items (i.e. avoiding washing/contact with stream water, taking an Onchocerciasis drug, use of chemical spray, and use of chemical treated bed net).

For each item, the correct response was given a score of 1 and incorrect responses assigned score of 0. The scores were then summed up to produce separate indices for each knowledge dimension, namely knowledge of symptoms, mode of transmissions, consequences and protective measures. The overall (comprehensive) knowledge score was computed by summing up all the aspects of knowledge which consisted of fourteen items, giving score values ranging from zero to fourteen. For purpose of standardizations and comparisons, all scales (sub-knowledge score and overall knowledge score) were rescaled to zero to ten values using Y = (X-X*min*)n/X range where Y is the rescaled variable, X is the original variable, X*min* is the minimum observed value on the original variable and X range is the difference between the maximum score and the minimum score on the original variable and n is the upper limit of the rescaled variable.

For over-all knowledge and its dimensions, the median value was used to slip the score into two; indicating adequate knowledge (above median value) and inadequate knowledge (median and below the median value).

#### Perceived risk of Onchocerciasis

Risk perception was measured using five items with a three point response (agree, disagree, neither/don’t know) format. For computing scores, each response of “agree” was recorded as 1; otherwise, zero points were recorded. The score was then summed up to produce risk perception score which consisted of five items, giving score values ranging from zero to five. For purpose of standardizations and comparisons, the score was rescaled to zero to ten values using Y = (X-X*min*)n/X range as knowledge above with the higher score indicating higher perceived risk of Onchocerciasis infection. The median value was used to divide the score in to high risk perception (above median value) and low risk perception (median and below median).

#### Onchocerciasis preventive practices

Onchocerciasis preventive practice was assessed using five items (i.e. avoiding washing with, swimming or touching river water, taking Onchocerciasis drugs during the campaign, sleeping under chemical treated bed net, covering the body fully with clothes while touching river water and spraying chemical insecticide). A “yes” preventive practice was considered if the respondent reported used at least one of preventive measures otherwise, coded as “0 and considered as no preventive practice”. A higher score reflects more preventive practices used at the household level.

For qualitative part of the study, a semi-structured guide was developed by reviewing pieces of literature related to the research objectives. The guide was developed by covering topics on Onchocerciasis its causes, mode of transmission, consequences and preventive measures. It was collected by trained master-level public health and social sciences professionals who were experienced in qualitative research and fluent in Afan Oromo. All discussions and interviews were recorded using a digital voice recorder and note-taking. A digital voice recorder was used to record all discussions and interviews. On average, KII have lasted for 1:20 hours, the FGDs for 1:45 hours and expert group discussion 1:35 hours.

### Data processing and analysis

The collected data were checked for completeness and cleaned, entered into Epi data manager version 4.0.2 and then exported to SPSS version 21 for analysis. Data were summarized and presented using descriptive statistics and in narrative texts, tables, and graphs. A logistic regression model was computed to see the association of independent variables with dependent variables (overall knowledge and preventive practices). Independent variables for overall knowledge were (Sociodemographic characteristics, study area, risk perception), likewise, for preventive practice (Sociodemographic characteristics, study area, risk perception, knowledge of symptoms, knowledge of mode of transmission, knowledge of consequences, knowledge of preventive measures and comprehensive knowledge). After doing a bivariable analysis, those independent variables with a p-value of less than 0.25 were moved to a multivariable analysis to control confounding factors. Finally, the 95% confidence interval and a p-value of less than 0.05 in multivariable logistic regression were used to ensure the significance of statistical association. Assumptions of logistic regression analysis including model fitness and multi-collinearity were checked and found to be fulfilled (**S3 Material**). Kruskal-Wallis and Mann-Whitney tests were used to compare median risk perception score by socio-demographic factors.

For qualitative part of the study, the audio data were transcribed verbatim and instantly translated to English following each interview and discussion. The coding and further analyses were assisted by Atlas.ti 7.1.4. The transcripts were read and re-read by the investigators who then allotted codes to all transcripts. An inductive approach of thematic analysis was used and the data were coded, categorized and thematized. Direct quotes were chosen to illustrate the interpretations and to help explain and clarify the quantitative results.

### Data quality control

Experienced data collectors who are fluent in local languages (Amharic and Afan Oromo) collected the data. Public health experts reviewed the tool to ensure its relevance and content validity, and it was translated into local language and back-translated into English to ensure consistency of the translations. Cronbach’s alpha coefficients were used to assess internal consistency of the instrument. The Cronbach’s alpha values for the items under each construct were 0.70 for risk perception and 0.609 for overall knowledge which is within acceptable range. Prior to the actual data collection, the tool was pretested to ensure its clarity and appropriateness in the local context, and necessary revisions were made. Data collectors received extensive training, and were strictly supervised during the data collection. STROBE checklist for observational study was used **(S4 Material).**

### Trustworthiness for the qualitative part

Various techniques were used to ensure the dependability, credibility, transferability, and conformability of the findings. First, a semi-structured guide was developed concerning the research questions and a diversified group of individuals were recruited. Second, data was triangulated by collecting through interviews and FGD from diversified study participants. Third, during and at the end of each interview and FGD, key points were summarized by the facilitators and the participants were requested to provide feedback and confirm whether the summaries reflected their ideas. Fourth, transcripts were shared with colleagues and feedback was considered. Fifth, an audit trail was done to confirm the study findings in order to ensure that the results were logical and data-driven. Sixth, to ensure transferability, the entire research process, methodology, study area, and results interpretation were all described in detail. Finally, trust was established with study participants by prolonged engagement.

## Results

### Socio-demographic characteristics of the respondents

In this study, 732 participants were involved, giving a response rate of 98.5%. Five hundred ninety five (81.3%) of the survey respondents were females. The respondents were predominantly farmers (94.1%) and Oromo (88.5%) [Table 1].

**Table 1:** Socio-demographic characteristics of participants in Jimma, October_2021.

Variables	Category	Frequency	Percentage
Study district	O/Neda	187	25.5
	O/Beyam	131	17.9
	Kersa	137	18.7
	Gomma	162	22.1
	Manna	115	15.7
Sex	Male	137	18.7
	Female	595	81.3
Age (year)	15–24	106	14.5
	25–34	194	26.5
	35–44	216	29.5
	45–54	117	16.0
	> = 55	99	13.5
Household role	Wife	576	78.7
	Husband	124	16.9
	Member	32	4.4
Marital status	Married	646	88.3
	Widowed	43	5.9
	Single	28	3.8
	Other ^a^	15	2.0
Educational status	No formal education	391	53.4
	Only able to read and write	18	2.5
	Primary education	277	37.8
	Secondary education	46	6.3
Religion	Muslim	638	87.2
	Orthodox	66	9.0
	Other ^b^	28	3.8
Ethnicity	Oromo	648	88.5
	Amhara	31	4.2
	Hadiya	25	3.4
	Other ^c^	28	3.8
Occupation	Farmer	689	94.1
	Other ^d^	43	5.8

^*a*^(6)separated, (9)divorced

^*b*^(26)protestant, (2) other

^*c*^(9)Dawuro,(4)Yem, (8)Kafa,(7)other

^*d*^(8)merchant or private business,(13) daily laborer, (1)NGO worker,(3)pastoralist

### Awareness and exposure to messages of Onchocerciasis

The majority of the respondents (84.3%) reported that they knew/had heard of Onchocerciasis (locally called “Oncho”). Qualitative evidence from community members showed that even though the Onchocerciasis campaign has been conducted for many years, there is a great knowledge gap about the disease among the communities. Some even believe Onchocerciasis is a drug, rather than a disease. *“Regarding Onchocerciasis*, *we usually create awareness during the campaign*. *But*, *people usually do not differentiate onchocerciasis from scabies*. *They come to our health post and ask Ivermectin for scabies” (Health extension worker*, *Kersa district*, *KII)*.

### Knowledge of symptoms and signs of Onchocerciasis

The majority of the respondents (84.3%) reported that they knew/had heard of Onchocerciasis. The commonest signs and symptoms of Onchocerciasis reported were intensive skin itching (69.4%) and skin rash (45.6%). Firm nodules on the skin and eye itching were reported by (6.4%) and (4.1%) of the participants respectively, while one fifth of respondents (20.2%) did not report any [Table 2].

**Table 2:** Knowledge of sign and symptoms of Onchocerciasis, October_2021.

Characteristics	Frequency (N = 732)	Percentage
Adequate knowledge	361	49.3
Intensive skin itching	508	69.4
Skin rash	334	45.6
Body ulceration	224	30.6
Leg swelling	102	13.9
Skin color change	77	10.5
Firm nodule on the skin	47	6.4
Eye itching	30	4.1
Abdominal pain	18	2.5
Inguinal swelling	13	1.7
Diarrhea	8	1.1
Vomiting	5	0.7
I don’t know	148	20.2
Other*	28	3.8

*(4)fever, (4)body swelling, (3)body weakness, (2)skin burning,(2)chills, (1)alopecia, (1)burns the body, (1)sweating, (1)wasting, (1)weaken the body, (1)eat within the body,(1)cough, (1)damages the body, (1) attack the body in a complicated way,(1)cut the breast.

The qualitative findings revealed the majority reported Onchocerciasis symptoms and signs such as intensive itching, watery discharge, skin irritation, rash and nodes, leg swelling, and leg wounds. They also pointed out that it leads to eye damage. *“The symptom of the disease is body itching that leads to ulcerations*, *watery discharges from muscle*, *this watery discharge is caused by a worm that bites when that worm reproduced in the human body*, *it causes skin swelling and finally destroys human eyes…”(P2*, *women*,*O/Beyam district*, *FGD)*

The study participants also mentioned that the disease has gastrointestinal symptoms such as abdominal cramps, diarrhoea and vomiting. *“I consider it as a problem in our community; because*, *if we didn’t take the oncho drugs it facilitates the growth of worms in the abdomen and result in abdominal cramps*, *vomiting and diarrhoea*. *Food will not stay in the abdomen for a while” (P9*, *school girl*, *Manna district*, *FGD)*.

### Knowledge of mode of transmissions and causes of Onchocerciasis

The study indicated that communities largely attributed Onchocerciasis causation to poor environmental sanitation (29.4%), poor personal hygiene (29.1%), exchange of clothes (25.7%) and direct contact (15.7%). Only 2.6% of respondents perceived that it is transmitted via contacts with stream water (e.g. swimming, washing, or other purposes), while 37.3% were unable to mention any risk for acquiring it. On the other hand, only 16.4% of the respondents were able to mention that the bite of blackfly causes Onchocerciasis, while 37.3% did not know how one could get this disease [Table 3].

**Table 3:** Perceived mode of transmissions and consequence of Onchocerciasis, October, 2021.

Characteristics	Frequency	Percentage
Adequate knowledge	131 215	17.9 29.4
Poor personal hygiene	213	29.1
Exchanging of clothes	188	25.7
Bite of blackfly	120	16.4
Direct contact with infected person	115	15.7
Eating contaminated food	50	6.8
Drinking unclean water	43	5.9
Swimming/washing with stream water/canal water	19	2.6
Washing with contaminated water	19	2.6
Worms infection	18	2.5
Lack of using toilet	18	2.5
Sexual intercourse	12	1.6
I don’t know	273	37.3
Other*	41	5.6
**Perceived consequences**		
Adequate knowledge	261	35.6
Stigma	402	54.9
Scar	343	46.9
Wasting	125	17.1
Death	67	9.2
Blindness	53	7.2
I don’t know	222	30.3
Other†	29	4.0

*(7)airborne, (6)drinking stream water, (5)walking barefoot, (5)malnutrition, (5) ("michii), (4)scarcity of food, (2)eating sweet food, (1)allergy, (1)stagnant water, (1)cattles dung, (1)cold, sweat, (1)drinking contaminated water,(1)eating meat and milk,(1)from bad and good smell things, (1)materials hygiene, (1)poverty, (1)raw fruit and vegetables, (1)takeoff sweat clothes and put on it, (1)playing in the dust,(1)washing by unclean water,(1)puberty,(1)exchange of soup, (1)good smell food,(1) dressing the cloth stayed in the sun

†(3)skin pigmentation, (2)skin color change to grey, (2)leg swelling, (1)bedridden and death, (1)body ulceration,(1)can disseminate to other bodies,(1)death and paralysis,(1)decrease appetite,(1)sickness,(1)diarrhea,(1)drying the body,(1)has no chronic consequence (harm),(1)it blacken body,(1)it burns body much,(1)it is damaging, (1)it makes hungry,(1)major organ damage,(1)make the skin brown,(1)prevent one from getting up from the ground, (1)scrotal swelling and death,(1)skin color change,(1)skin disease,(1)stunting, (1)un-healing skin pigmentation, (1) wound).

### Causes of Onchocerciasis

The qualitative findings revealed that the majority of study participants repeatedly mentioned that the cause of Onchocerciasis is poor environmental sanitation and personal hygiene.

*“I think Onchocerciasis has resulted from poor sanitation”* (*P5*, *O/Neda district, FGD)*

*“People know that Onchocerciasis is caused by blackfly. The fly usually breeds around water*. *It is transmitted to people when people touch the water or walk barefoot. It can be prevented by wearing shoes” (KII, Ganda Leader, O/ Beyam district).*

However, some participants mentioned that Onchocerciasis is caused by mosquito bites.

*“This disease is caused by a mosquito living around the rivers*. *Our Ganda is among the two identified kebeles in Gomma district in which this mosquito is living.” (KII, Vice Ganda leader, Gomma district)*

Some also mentioned that invisible flies, germs, worms living in the water and parasite are the causes of Onchocerciasis. This study also found that walking barefoot, using running water and swimming are known to be risk factors for Onchocerciasis.

*“I do not know from where oncho disease comes from; however*, *I heard from health workers that a worm causes the disease” (FGD, P4, Women, Gomma district).*

*“……Oncho is also a fear for our health because we are using running water for many purposes like washing our clothes and our children swimming in those rivers”* (*FGD, P3, Women, Gomma district*)

### Modes of transmission of Onchocerciasis

Qualitative evidence revealed that the majority of the study participants stated that contact is the main mode of transmission of Onchocerciasis, and one participant of FGD (school boy), mentioned sexual contact as the mode of transmission. *“…*. *Sharing clothes with an infected individual can transmit oncho from an infected individual to the healthy one*.*” (P1*, *school boy*, *FGD*, *O/Neda district)*

The study participants also stated that bites while washing clothes near a river or washing legs near water is one of the modes of transmission of Onchocerciasis.

*“…It is caused by a fly that lives near the river*. *If you wash your leg or clothes near the water, it may bite you, causing you to contract the illness” (FGD, P9, Women, Gomma district)*

Regarding the consequences of infections with Onchocerciasis, 46.9% of the respondents mentioned that body scarring is one of its major consequences and 7.2% reported blindness; yet about one third (30.3%) were unable to report any of the consequences [Table 3].

Qualitative data revealed that the majority of the participants knew that Onchocerciasis affects the vision of individuals and leads to blindness. *“Oncho causes swelling and wounds of legs and blindness……*.*” (KII*, *Vice ganda leader*, *Gomma district)*.

### Knowledge on preventive methods of Onchocerciasis

Respondents were asked about their awareness and perceptions about how one can prevent Onchocerciasis. Accordingly, the respondents frequently mentioned maintaining personal hygiene (51.4%), environmental sanitation (43.7%) and taking the drug during a campaign (43.7%) as preventive measures. Only (5.6%) of study participants indicated that avoiding contact with stream water helps to prevent Onchocerciasis [Table 4].

**Table 4:** Knowledge on preventive methods of Onchocerciasis, October_2021.

Characteristics	Frequency	Percentage
Adequate knowledge	347	47.4
Keeping personal hygiene	376	51.4
Keeping environmental sanitation	320	43.7
Taking oncho drug during campaign	320	43.7
Avoiding exchange of clothes	172	23.5
Avoiding washing/contact with stream water	41	5.6
Using chemical treated bed net	9	1.2
Avoiding walking bare footed	7	1.0
Chemical spray	6	0.8
I don’t know	138	18.9
Other*	46	6.3

*(5)taking other medication, (4)eating balanced food, (4) traditional medicine, (3)taking medication, (3) clean food, (3) going to health care institution, (2) wearing clean clothes, (2) eating food well, (2) using toilet, (1)accepting HWs advice, (1) being at far from oncho patients,(1)changing clothes, (1) draining accumulated water,(1) eating meat and cabbage, (1) eating variety of food,(1) eating what is not clean,(1) fleeing the infected person,(1)isolation, (1) Keeping materials’ hygiene,(1) removing stagnant water,(1) taking allergy ("michii")medication, (1) using solid and liquid waste management, (1)vaccination,(1) wash thoroughly frequently,(1) wash with hot water in the morning, (1)Washing clothes, (1)washing with running water,(1) washing with salty water,(1)washing with soap and water.

On the other hand, the majority of study participants highlighted that treatment of the cases and drug distribution campaigns as prevention and control methods of Onchocerciasis.

*“…If the drug is received well*, *it can be prevented*. *But the drug will not kill the worm, rather it makes the worm infertile and not to transmit the disease. (FGD, P7, women, O/Beyam district)”*

Some of the participants explained that avoiding sharing clothes, practicing good hygiene and wearing shoes helps to prevent onchocerciasis disease. KII, vice Kebele Leader, Gomma District, for instance, stated: *“…*. *we can prevent Onchocerciasis by avoiding sharing clothes and by keeping our hygiene*. *We can also prevent it by taking its drug*.*”*

### Risk perception towards Onchocerciasis

Five hundred fifty four (75.7%) of the respondents perceived that Onchocerciasis is a severe disease. Although 44.7% and 42.8% respectively perceived that swimming/washing clothes near streams and living near running water risk exposure to Onchocerciasis, about one third perceived themselves and their families as vulnerable to it [Table 5].

**Table 5:** Risk perception towards Onchocerciasis, October_2021.

Items	Agree N(%)	Disagree N(%)	Don’t know N(%)
People who frequently touch running water (washing, swimming, getting in) are at risk of Onchocerciasis	327(44.7)	256(35.0)	149(20.4)
I am at risk of getting Onchocerciasis	227(31.0)	407(55.6)	98(13.4)
A person or family living near running water or rivers is at high risk of getting oncho	313(42.8)	267(36.5)	152(20.8)
My families are at risk of Onchocerciasis	228(31.1)	409(55.9)	95(13.0)
Oncho is a severe disease	554(75.7)	78(10.7)	100(13.7)

### Median risk perception score comparison

The median risk perception score was varied significantly between the groups by educational status (p = 0.043), study district (p = 0.047) and age category (p = 0.018) according to the Kruskal-Wallis test [Table 6].

**Table 6:** Kruskal-Wallis Test and Mann-Whitney U Test of risk perception score with socio-demographic characteristics, October_2021.

Parameters	Frequency (%)	Chi-square value	P-value
**Educational level**			
No formal education	391(53.4)	8.15	0.043**
Only able to read and write	18(2.5)		
Primary education	277(37.8)		
Secondary education	46(6.3)		
**Study district**			
O/Nada	187(25.5)	9.61	0.047**
Gomma	162(22.1)		
O/Beyam	131(17.9)		
Kersa	137(18.7)		
Manna	115(15.7)		
**Age category (year)**			
15–24	106(14.5)	11.85	0.018**
25–34	194(26.5)		
35–44	216(29.5)		
45–54	117(16)		
> = 55	99(13.5)		
**Ethnicity**			
Oromo	648(88.5)	4.71	0.194
Amhara	31(4.2)		
Hadiya	25(3.4)		
Other	28(3.8)		
**Religion**			
Muslim	638(87.2)	5.39	0.068
Orthodox	66(9)		
Other	28(3.8)		
Mann-Whitney U Test			
**Sex**		Z value	
Male	137(18.7)	-0.711	0.477
Female	595(81.3)		

**Value statistically significant

### Preventive practices of Onchocerciasis

Table 7 shows survey responses about Onchocerciasis prevention practices. Accordingly, taking the Onchocerciasis drug during the campaign (41.7%) and avoiding bathing, touching, or washing with river water (8.9%) reported most frequently. However, the majority of respondents stated that their Onchocerciasis prevention practices included maintaining personal hygiene (55.9%) and environmental sanitation (46%). The overall percentage of respondents practicing at least one preventive measure of Onchocerciasis was 343(46.9%) [Table 7].

**Table 7:** Preventive practices of Onchocerciasis of the respondents, October_2021.

Characteristics	Frequency (n = yes)	Percentage
Practice at least one	343	46.9
Keeping personal hygiene	409	55.9
Keeping environmental sanitation	337	46.0
Taking oncho drug during the campaign	305	41.7
Avoiding sharing clothes	174	23.8
Doing nothing	111	15.2
Avoiding washing with, swimming or touching running water	65	8.9
Sleeping under chemical treated bed net	15	2.0
Covering the body fully with clothes while touching river water	7	1.0
Spraying chemical	6	0.8
Avoiding walking barefoot	6	0.8
We cannot avoid	4	0.5
I don’t know	66	9.0
Other*	32	4.3

*(9)isolation, (3)eating balanced diet, (2)avoidance of living near swampy areas, (2)using medical soap, (2)using toilet,(1) applying lemon to our body, (1)avoid touching or using lake water,(1) Avoiding Physical contact,(1) draining accumulated water, waste management,(1) drinking clean water, (1) keeping food hygiene,(1) keeping materials hygiene, (1)traditional medicine, (1)using mask, (1)washing clothes, (1)preventing contacting each other and hygiene of cloth

### Overall knowledge and risk perception

**Fig 1:**
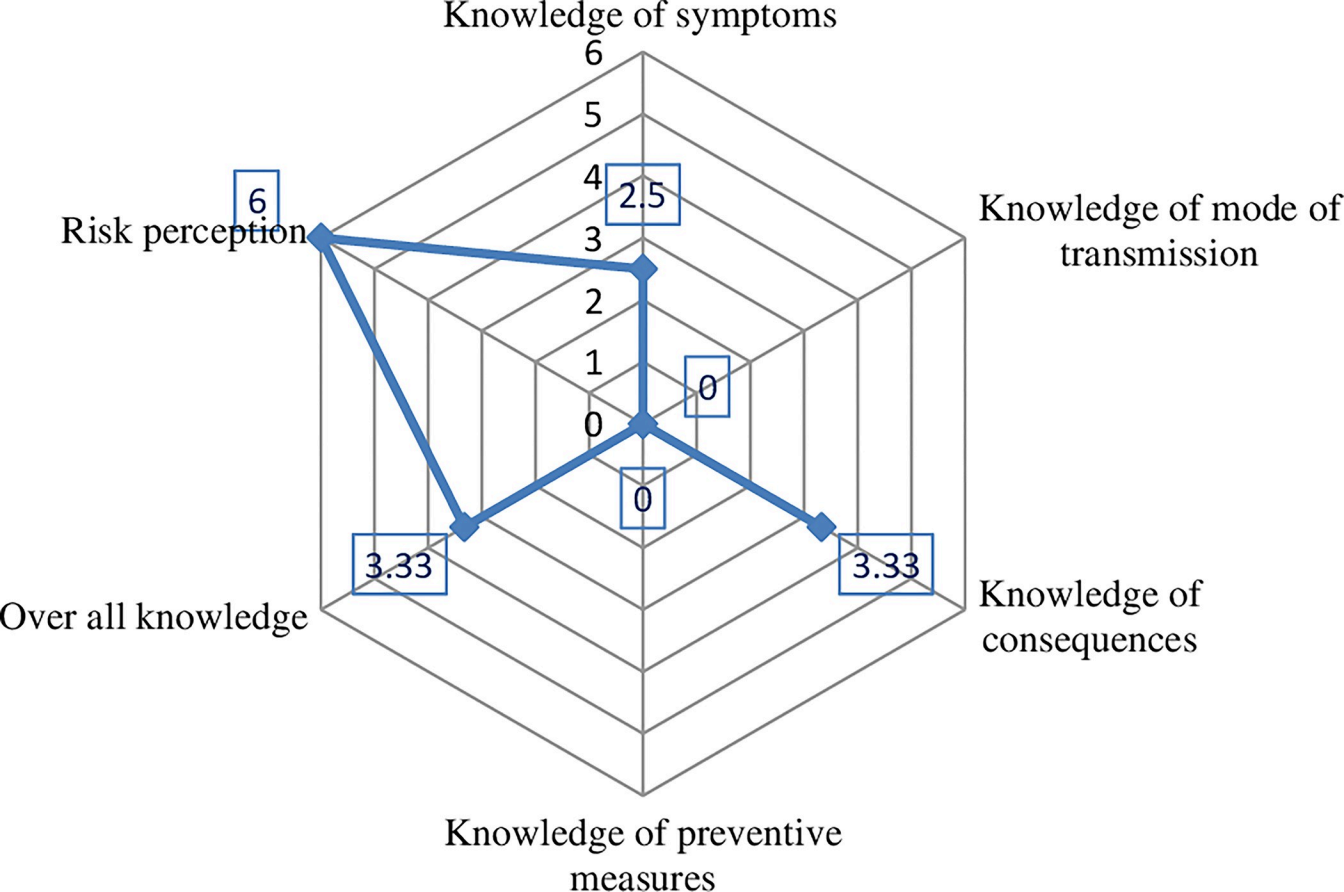
Median score on overall knowledge, its dimension and risk perception towards to Onchocerciasis.

After the score was computed and rescaled to standardize (0–10 score), the median value used to split each score and the result is plotted in radar chart [Fig 1]. Consequently, lowest median score was recorded for knowledge of mode of transmission and preventive measures while the highest was recorded for risk perception. Median score was used to define adequate multidimensional knowledge and high risk perception giving prevalence of 357(48.8%) and 137(18.7%) respectively.

### Overall knowledge, risk perception and preventive practice by districts

**Fig 2:**
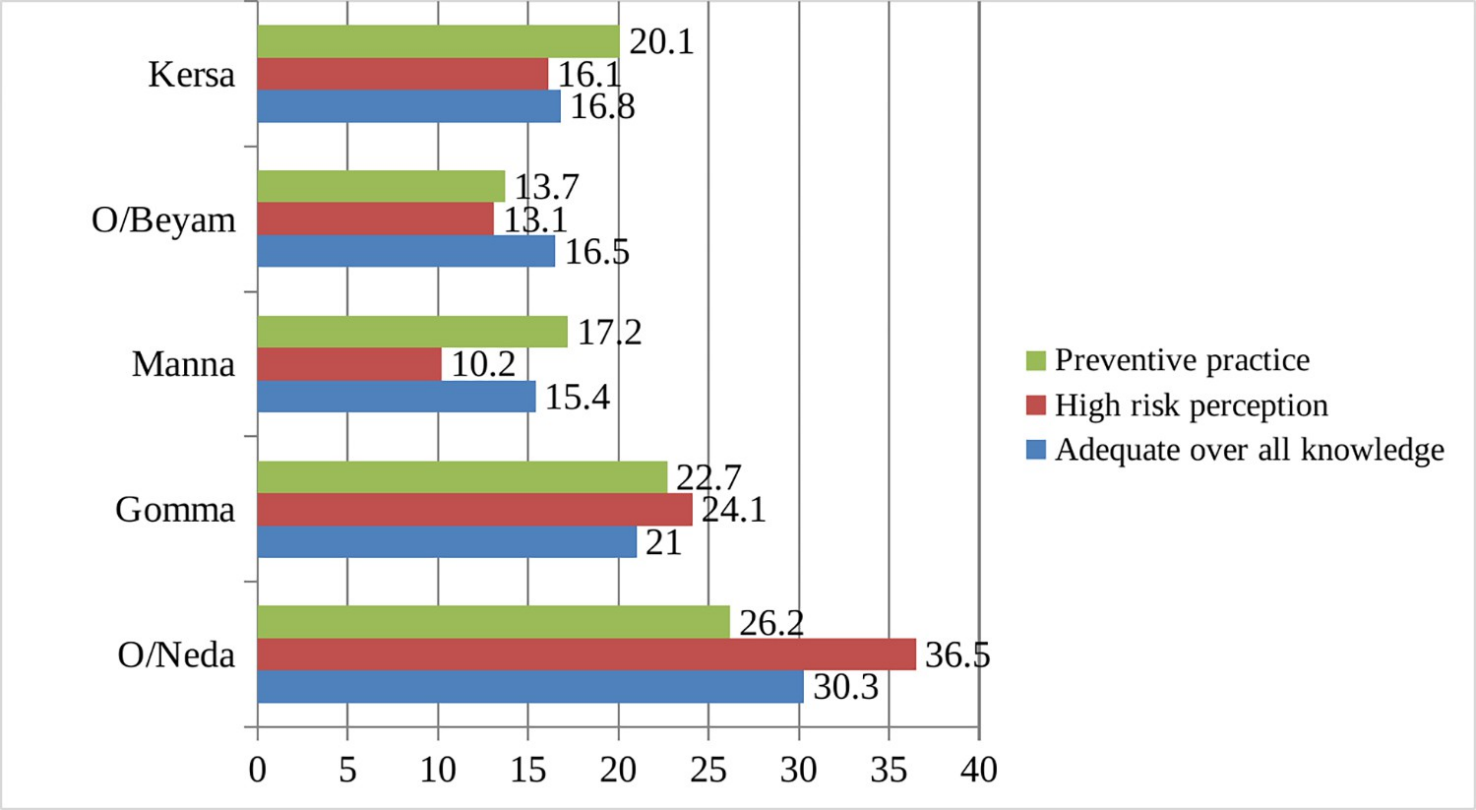
Percentage of overall knowledge, risk perception and preventive practice by districts.

Fig 2 illustrates comparison of three important aspects of the measurement: overall knowledge of preventive measures, risk perception and preventive practices after the score was standardized. The O/Neda district participants showed the highest levels in three of them, whereas high risk perception and over all knowledge is lowest in Manna district. Use of preventive practices was lowest in O/Beyam district [Fig 2].

### Factors associated with overall knowledge

Table 8 shows factors associated with overall knowledge about Onchocerciasis. Consequently, eight variables (district, sex, role in household, educational level, ethnicity, religion, age, and risk perception) showed evidence of association with the overall knowledge at a p-value <0.25. From those variables, only risk perception was statistically significant with overall knowledge. Participants who had high risk perception were 1.95 times more likely to have adequate overall knowledge of the disease, compared to participants with low risk perception (AOR = 1.95 95%CI (1.32, 2.8)) [Table 8].

**Table 8:** Factors related to overall knowledge towards Onchocerciasis in Jimma zone, October_2021.

Variables	Category	Overall knowledge					
		In adequate n (%)	Adequate n (%)	P- value	COR(95%CI)	P-value	AOR (95%CI)
Study district	O/Nada	79(21.1)	187(25.5)	0.093	1.45(0.94,2.38)	0.206	**
	Gomma	87(23.2)	162(22.1)	0.802	0.94(0.58,1.52)	0.183	**
	O/Beyam	72(19.2)	131(17.9)	0.662	0.89(0.54,1.48)	0.879	**
	Kersa	77(20.5)	137(18.7)	0.522	0.85(0.52,1.39)	0.936	**
	Manna	60(16.0)	115(15.7)	1	1	1	
Sex	male	64(46.7)	73(53.3)	0.242	1.25(0.86,1.81)	0.902	**
	female	311(52.3)	284(47.7)	1	1	1	1
Age (years)	15–24	48(45.3)	58(54.7)	0.142	1.51(0.87,2.62)	0.254	**
	25–34	98(50.5)	96(49.5)	0.414	1.22(0.75,1.99)	0.434	**
	35–44	113(52.3)	103(47.7)	0.593	1.14(0.71,1.84)	0.785	**
	45–54	61(52.1)	56(47.9)	0.616	1.15(0.67,1.96)	0.796	**
	> = 54	55(55.6)	44(44.4)	1	1	1	1
Educational status	No formal education	207(52.9)	184(47.1)	1	1	1	1
	Only able to read and write	7(38.9)	11(61.1)	0.249	1.76(0.67,4.65)	0.467	**
	Primary	141(50.9)	136(49.1)	0.603	1.08(0.79,1.47)	0.470	**
	Secondary	20(43.5)	26(56.5)	0.226	1.46(0.79,2.70)	0.172	**
Role in the household	housewife	304(52.8)	272(47.2	1	1	1	1
	Husband	59(47.6)	65(52.4)	0.294	1.23(0.84,1.82)	0.491	**
	member	12(37.5)	20(62.5)	0.097	1.86(0.89,3.88)	0.281	**
Religion	Muslim	320(50.2)	318(49.8)	1	1	1	1
	Orthodox	40(60.6)	26(39.4)	0.108	0.65(0.39,1.09)	0.789	**
	Other	15(53.6)	13(46.4)	0.724	0.87(0.41,1.86)	0.279	**
Ethnicity	Oromo	324(50)	324(50)	1	1	1	1
	Amhara	18(58.1)	13(41.9)	0.382	0.72(0.35,1.49)	0.587	**
	Hadiya	16(64)	9(36.0)	0.175	0.56(0.25,1.29)	0.141	**
	Other	17(60.7)	11(39.3)	0.270	0.65(0.29,1.40)		**
Risk Perception	low	323(54.3)	272(45.7)	1	1	1	1
	high	52(38.0)	85(62.0)	0.001	1.94(1.33,2.84)	0.001*	1.95(1.32,2.8)

*: value statistically significant

**: value statistically not significant 1: reference

### Factors associated with preventive practices

Table 9 shows factors associated with preventive practices towards Onchocerciasis. Consequently, twelve variables (study district, age, marital status, educational level, religion, occupation, knowledge of mode of transmission, knowledge of consequences, knowledge of preventive measures, knowledge of sign and symptoms, comprehensive knowledge and risk perception) showed evidence of association with the outcome (preventive practices) at a p-value <0.25. Of those variables, knowledge of mode of transmission, knowledge of consequences and knowledge of preventive measures were statistically significant with preventive practices. Participants who had knowledge on mode of transmission were 2.64 times more likely to engage in preventive practices than participants without that knowledge (AOR = 2.64 95%CI (1.44, 4.85)). Participants who had knowledge on consequences of Onchocerciasis were 2.12 times more likely to engage in preventive practices than participants without that knowledge (AOR = 2.12 95%CI (1.21, 3.72)). Furthermore, participants who had knowledge of preventive measures were 15.65 times more likely to engage in preventive practices than their counter parts without that knowledge (AOR = 15.65 95% CI (10.1, 24.2)) [Table 9].

**Table 9:** Factors associated with preventive practices towards Onchocerciasis in Jimma zone, October_2021.

Variables	Category	preventive practice					
		No	yes	P- value	COR (95%CI)	P-value	AOR (95%CI)
Study district	O/Nada	97(51.9)	90(48.1)	1	1	1	
	Gomma	84(51.9)	78(48.1)	0.997	1(0.66, 1.53)	0.530	**
	O/Beyam	84(64.1)	47(35.9)	0.030	0.6(0.38,0.95)	0.606	**
	Kersa	68(49.6)	69(50.4)	0.691	1.09(0.70,1.70)	0.402	**
	Manna	56(53.1)	59(46.9)	0.592	1.13(0.71,1.81)	0.170	**
Age (years)	15–24	50(47.2)	56(52.8)	0.103	1.58(0.91,2.75)	0.622	**
	25–34	107(55.2)	87(44.8)	0.576	1.15(0.71,1.87)	0.822	**
	35–44	116(53.7)	100(46.3)	0.419	1.22(0.75,1.97)	0.669	**
	45–54	58(49.6)	59(50.4)	0.186	1.44(0.84, 2.47)	0.297	**
	> = 54	58(58)	41(42.0)	1	1	1	
Occupation	farmer	372(54)	317(46.0)	1	1	1	1
	other	17(39.5)	26(60.5)	0.069	1.79(0.96,3.37)	0.245	**
Marital status	married	343(53.1)	303(46.9)	1	1	1	1
	widowed	28(65.1)	15(34.9)	0.129	0.61(0.32, 1.157)	0.457	**
	single	11(39.3)	17(60.7)	0.157	1.75(0.81,3.79)	0.705	**
	other	7(46.7)	8(53.3)	0.623	1.29(0.46,3.61)	0.511	**
Educational status	No formal education	216(55.2)	175(44.8)		1	1	1
	Only able to read and write	9(50)	9(50.0)	0.662	1.23(0.48,3.18)	0.616	**
	Primary	146(52.7)	131(47.3)	0.517	1.11(0.81,1.51)	0.268	**
	Secondary	18(39.1)	28(60.9)	0.041	1.92(1.03,3.59)	0.955	**
Religion	Muslim	333(52.2)	305(47.8)	1	1	1	1
	Orthodox	40(60.6)	26(39.4)	0.194	0.71(0.42,1.19)	0.320	**
	Other	16(57.1)	12(42.9)	0.608	0.82(0.38,1.75)	0.437	**
Risk perception	low	332(55.8)	263(44.2)	1	1	1	1
	high	57(41.6)	80(58.4)	0.003	1.77(1.22,2.58)	0.338	**
Knowledge of mode of transmission	no	359(59.7)	242(40.3)	1	1	1	1
	yes	30(22.9)	101(77.1)	<0.001	4.99(3.22,7.75)	0.002*	2.64(1.44,4.85)
Knowledge of consequences	no	166(78.7)	45(21.3)	1	1	1	1
	yes	223(42.8)	298(57.2)	<0.001	4.93(3.39,7.15)	0.008*	2.12(1.21, 3.72).
Knowledge of Preventive measures	No	322(83.6)	63(16.4)	1	1	1	1
	yes	67(19.3)	280(80.7)	<0.001	21.36(14.6,31.2)	<0.001*	15.65(10.1,24.2)
Knowledge of sign and symptoms	No	133(34.5)	252(65.5)	1	1	1	1
	yes	41(11.8)	306(88.2)	<0.001	3.83(2.59,5.64)	0.941	**
Over all knowledge	inadequate	281(74.9)	94(25.1)	1	1	1	1
	adequate	108(30.3)	249(69.7)	<0.001	6.89(4.98,9.53)	0.266	**

*value statistically significant not

** value statistically not significant; 1: reference

## Discussion

Onchocerciasis is one of the NTDs Ethiopia has targeted to eliminate [11]. Accurate community understanding of the disease and its mode of transmission and exercising appropriate preventive practices are crucial to support the elimination prospects of this disease. In this study we assessed community knowledge, risk perception, and use of preventive practices regarding Onchocerciasis using a mixed method study in Jimma zone. The findings revealed that the vast majority of people (84.3%) in the study communities had ever heard of Onchocerciasis (locally called "Oncho"). However, there is a great knowledge gap about the disease among the community. There was some degree of misinterpretation and confusion in distinguishing Onchocerciasis from its drugs–some believe Onchocerciasis is a ‘drug’ rather than a disease, and vice versa. In this context, ‘Oncho’ referred to a drug being distributed by the community volunteers. This local meaning and interpretation of the disease/drug will have a significant implication for health education activities, especially for health communications message development and delivery, which must be locally sensitive. Helping people to recognize the disease by its symptoms and manifestation is one of the goals of public health education because it encourages people to seek care early thereby contributing to prevention and control efforts [32,57].

Our finding revealed that there were insufficient levels of comprehensive knowledge, risk perception, and preventive practice towards Onchocerciasis. High risk perception was significantly associated with overall knowledge, likewise knowledge of mode of transmission knowledge of consequences and knowledge of preventive measures were significantly associated with preventive practice.

This study revealed that community knowledge of classic signs and symptoms of Onchocerciasis was generally insufficient as only half of the respondents were able to adequately recognize Onchocerciasis by its collective symptoms. Indeed, symptoms of Onchocerciasis such as skin itching (69.4%) and skin rash (45.6%) were relatively better known in the study community which is comparable with some previous studies [2,50]. The possible reasons for the similarities could be due to similarity in the study population, study design and exposure to health information. In elimination context, there is compelling evidence that people’s ability to recognize the target disease often drops due to reduced prevalence and visibility of the disease [58]. The national Onchocerciasis elimination program required to note this issue as it could be a prominent challenge to sustained community engagement in Onchocerciasis control and elimination programmes.

Community knowledge regarding how the disease could spread or how one could acquire it is an important health literacy aspect as far as Onchocerciasis is concerned. In our study, knowledge of Onchocerciasis transmission routes was quite low, with only 16.4% of survey respondents able to correctly identify the blackfly bite as the mode of transmission of Onchocerciasis. Despite a community- based Onchocerciasis program having been conducted for decades in the study community, the level of public awareness about the causative agent of Onchocerciasis is still unacceptably low. It may be important to evaluate the content of communication messages being addressed to the community with the aim to optimize and ensure the appropriateness and completeness of such message contents. Indeed, some earlier studies reported comparable finding [16,47,59]. However, our finding was higher than a study reported in South Western Nigeria (2.4%) [60]. This could be due to differences in sample size, study period, access to health information and geographical location. On the other hand, our result was much lower than the study done in Northwest Ethiopia 69.4% [2] and 73.8% in Gesha town, Southwest Ethiopia [49]. The possible reasons could be due to differences in study participants, study area and sociocultural differences. For instance, the majority of our participants were women, who are more likely to engage in indoor activities and are less likely to be exposed to information, which may have an impact on their knowledge of the route of transmission. Moreover, consistent with the other finding [61], our result showed a wide range of misconceptions about the route of Onchocerciasis transmission where significant portion of the study community attributed poor hygiene (29.1%) and exchange of clothes (25.7%) to causation of Onchocerciasis. While linking poor hygiene and exchange of clothes to disease transmission would be something positive, it is important for people to establish the proper linkage of risk factors to specific diseases. This implies it is critical to raise community awareness in order to promote an appropriate and broader understanding among the public regarding the role of blackfly and the risks of living its ecosystem.

Another community knowledge gap in this study was about consequences of Onchocerciasis. In our study, a significant number of the study population was not able to recognize the consequences of Onchocerciasis such as skin scarring, blindness, and associated social stigma, which is supported by earlier findings [60,62]. In this study, only 7.2% of respondents pointed out that Onchocerciasis could lead to blindness, which was slightly higher than a study conducted in South Western Nigeria [60], but, it was much lower than study done in the in Democratic Republic of Congo (90.7%) [63] and elsewhere in Ethiopia (85.9%) [64]. This gap might be caused by difference in study participant’s educational status, study period as well as difference in access to health education information. Participants in our qualitative study appeared to have better awareness that Onchocerciasis leads to vision problems. This could be due to differences in sample selection and participants’ awareness level.

Even though MDA has been performed, with good coverage reports, for more than 15 years in Ethiopia and the study community [32], it is surprising that only 47.4% had adequate knowledge on Onchocerciasis preventive measures which was comparable with other finding [2]. Moreover, only two fifth indicated use of drug Ivermectin during campaign as the preventive measure which was consistent with previous studies [2,59,63]. The possible reason for similarity could be due to similarity in control programmes, study design and study population.

More than half of our study participants had the misconception that hygiene is a preventive measure. Only 5.6% of the participants reported avoiding contact with stream water as a preventive measure, which contrasts a study done in Giesha Kebele river basin, Oromia region, Ethiopia (46.2%) [24]. The probable reason could be differences in access to health information, study period, and programme implementation.

The study showed that high risk perception towards Onchocerciasis was significantly associated with better comprehensive knowledge about the disease. Higher risk perception may motivate people to seek and acquire more information, which enabled better understanding about the disease. Furthermore, higher risk perception determines the correct assessment of disease seriousness, which helps people adopt the right behaviors to combat it.

According to our study, only 18.7% of the participants had high risk perception towards Onchocerciasis infection which was quite low, implying that people believe their likelihood of acquiring Onchocerciasis is minimal. This finding was lower than study reported from Democratic Republic of Congo [63]. The probable reason could be differences in level of awareness, access to health education information, educational status, study period and sample size. A higher risk perception influences the adoption of effective disease-prevention behaviors, as perceived severity is more important than the actual severity of the disease in driving preventive practices. In fact, risk perception is complex and is greatly influenced by a wide range of factors, including general esteem, beliefs, trust, perceptions, contexts, geographic locations, people’s daily lives, socio-cultural systems, and environment [65,66].

The Kruskal-Wallis tests revealed that there were significant differences between the groups in terms of educational status, study district and age category in their risk perception towards Onchocerciasis. This might be as a result of the independent relationship between educational status and knowledge which can influence risk perception towards Onchocerciasis. Additionally, older individuals may have longer time exposure to Onchocerciasis than younger individuals, which may also affect their risk perception. Finally, there might be variations in each district’s Onchocerciasis control programme implementation and accessibility to health education, which may have an impact on people’s risk perception.

The study revealed the prevalence of adequate comprehensive knowledge on Onchocerciasis was 48.8%. This demonstrated that almost half of respondents lack comprehensive knowledge, which contributed to the persistence of Onchocerciasis infection in the community, as Onchocerciasis knowledge significantly, affects its prevalence of infection [67]. This finding was in line with studies conducted in Selamogo woreda, South West Ethiopia(51.7%) [68] and Quara, North West Ethiopia (45.5%) [2]. On the other hand, this study was lower than study conducted in Bench Maji zone, Southwest Ethiopia (67%) [50] and Giesha Kebele river basin, Oromia region, Ethiopia (75.9%) [24]. The possible reasons for inconsistency include differences in operational definitions used, study period, access to health education information, and study setting. Besides, this study was higher than study done in in Illu Ababora, South West Ethiopia (34%) [69] and Jimma 11.2% [16]. This could be due to differences in sample size, operational definition used, study period and intervention measures.

Our finding revealed that the proportion of at least one preventive practice used was 46.9%, and it is remarkable that almost half of our study subjects did not practice any method for preventing the disease. The prevention practices were consistent with comprehensive knowledge of Onchocerciasis (48.8%). The preventive practices result agreed with a study conducted in Tombel District, Cameroon [70]. The probable reason could be due to similar population, design and intervention measures. On the contrary, the finding was higher than study conducted in Selamogo (14.3%) [68] and in Illu Ababora, South West Ethiopia (22.7%) [69]. Surprisingly, a 2015 study in the Jimma revealed that none of the community member had preventive practices against Onchocerciasis [16]. Likewise, this study finding was lower than the study conducted in Bench Maji zone (60.4%) [50]. The reason could be due to differences in study population, educational status, operational definition applied, access to health information and variations in control programme implementation.

Multivariable analyses revealed that knowledge of mode of transmission, consequences and preventive measures were significantly associated with higher adoption of Onchocerciasis preventive practices. This may be due to the fact that people who were aware of the disease’s mode of transmission, consequences, and preventive measures were more likely to adhere to its preventive measures, and it emphasizes the significance of health education as a means of encouraging proper community behavior in places where the disease is endemic. Knowledge of the disease has been one of the most crucial criteria for local campaigns to eradicate onchocerciasis and raise awareness of the issue [32,47,49]. Lack of knowledge is one of the challenges to eradicating onchocerciasis, as it is a critical component of effective prevention strategies [2,32]. For any disease control efforts, good understanding of the condition is required and misconceptions obstruct community compliance and participation in intervention programs [23]. Thus, our findings highlight the need for tailored, context-specific behavior change communications aimed at dispelling misconceptions in order to control and eradicate Onchocerciasis in the community.

## Conclusion

Most of the respondents had heard about Onchocerciasis. However, only one sixth of the individuals reported perceived risk of it. More than half of the participants did not practice at least one recommended preventive measure, and also lack adequate knowledge of Onchocerciasis. Furthermore, risk perception was significantly associated with comprehensive knowledge of Onchocerciasis, whereas knowledge of mode of transmission, consequences and preventive measures were significantly associated with Onchocerciasis preventive practice. It is important to note that correct perceptions and proper understanding of this disease was generally lacking or inadequate in these endemic communities. It has been shown that knowledge is a key component of effective prevention strategies, as it is a necessary condition for behavior change to occur. In particular, it is important to build community knowledge and proper understanding of the mode of transmission (especially the role of blackfly), consequences and the prevention measures. Thus, our findings highlight the need for strengthening tailored and context-specific behavior change communications by integrating them into routine Onchocerciasis control and elimination programs. The findings would be helpful in creating community education and awareness raising efforts and would support ongoing onchocerciasis prevention, control, and elimination efforts in Ethiopia and other similar areas. In this regard, ongoing regular MDA campaigns for Onchocerciasis chemotherapy would be an ideal opportunity for integration of effective social and behavioral change communications elements to improve and sustain the community’s appropriate understanding and practices supporting Onchocerciasis control and elimination programs.

### Strength and limitation of the study

This study employed a mixed method approach which enabled us to gain deeper insight about the study objective. The sample size, both for the survey and qualitative, was relatively large which can contribute to the precision of the estimates. A potential study limitation could be social desirability bias.

## Supporting information

S1 Material The figure summarizing our study design on community’s knowledge, perceptions and preventive practices on Onchocerciasis in Jimma zone, Ethiopia. (DOCX)

S2 Material Table on the background characteristics of the key informants and focus group discussion participants. (DOCX)

S3 Material Assumptions of logistic regression knowledge (model fitness and multi-collinearity were checked). (DOCX)

S4 Material Filled STROBE Statement—Checklist of items that should be included in reports of observational studies. (DOCX)

S5 Material Oncho_SPSS file_2021.sav; Data used in the analysis (opened with SPSS software). (SAV)
