# Supplementary material for: Community’s knowledge, perceptions and preventive practices on Onchocerciasis in Jimma zone, Ethiopia, formative mixed study
Source: PLoS Negl Trop Dis. 2024 Mar 13;18(3):e0011995. doi: 10.1371/journal.pntd.0011995 (PMC10936768; doi:10.1371/journal.pntd.0011995)
Supplement: S1 Material — (DOCX) [file pntd.0011995.s001.docx]

**Supplementary material 1**

**Fig: The figure summarizing our study design on community’s knowledge, perceptions and preventive practices on Onchocerciasis in Jimma zone, Ethiopia**
