# Supplementary material for: Community’s knowledge, perceptions and preventive practices on Onchocerciasis in Jimma zone, Ethiopia, formative mixed study
Source: PLoS Negl Trop Dis. 2024 Mar 13;18(3):e0011995. doi: 10.1371/journal.pntd.0011995 (PMC10936768; doi:10.1371/journal.pntd.0011995)
Supplement: S2 Material — (DOCX) [file pntd.0011995.s002.docx]

**Supplementary material 2**

**Table:** Background characteristics of the key informants and focus group discussion participants

| **Background characteristics of the KII participants** | | | | |
| --- | --- | --- | --- | --- |
| S/N | Program work in | | Educational status | Experience |
| 1 | Community drug distributors | | No formal education | More than 10 years |
| 2 | Vice ganda leader | | Grade 6 | 2 years |
| 3 | Community drug distributors | | Can’t read and write | 7 years |
| 4 | Ganda leader | | Grade 6 | 3 years |
| **Background characteristics of the FGD participants** | | | | |
| Group | Participants | Number of participants | Age range | Education |
| 1 | School boys | 9 | 14 to 25 | From grade 7 to 10 |
| 2 | School girls | 11 | 13 to 16 | From 5 to 9 |
| 3 | Male community member | 8 | 18 to 60 | No education to grade 10 |
| 4 | School boys | 7 | 17 to 18 | 6 to 10 |
| 5 | Women community member | 10 | 32 to 45 | No formal education to grade 6 |
| 6 | Women community member | 11 | 19 to 40 | No formal education to grade 10 |
