# Supplementary material for: Community’s knowledge, perceptions and preventive practices on Onchocerciasis in Jimma zone, Ethiopia, formative mixed study
Source: PLoS Negl Trop Dis. 2024 Mar 13;18(3):e0011995. doi: 10.1371/journal.pntd.0011995 (PMC10936768; doi:10.1371/journal.pntd.0011995)
Supplement: S3 Material — (DOCX) [file pntd.0011995.s003.docx]

**Supplementary material 3**

Assumptions of logistic regression knowledge (model fitness and multi collinearity were checked)

**For overall knowledge**

| **Hosmer and Lemeshow Test for model fitness (0.881)** | | | |
| --- | --- | --- | --- |
| Step | Chi-square | df | Sig. |
| 1 | 3.727 | 8 | **0.881** |

**Table: Multicollinearity test of factors associated with overall knowledge**

| **Coefficients^a^** | | | | | | | | |
| --- | --- | --- | --- | --- | --- | --- | --- | --- |
| Model | | Unstandardized Coefficients | | Standardized Coefficients | t | Sig. | Collinearity Statistics | |
|  |  | B | Std. Error | Beta |  |  | Tolerance | VIF |
|  | (Constant) | .523 | .179 |  | 2.918 | .004 |  |  |
|  | Study district | -.020 | .013 | -.057 | -1.511 | .131 | .950 | 1.053 |
|  | sex | -.002 | .060 | -.002 | -.035 | .972 | .607 | 1.648 |
|  | Role in the household | .071 | .045 | .075 | 1.567 | .118 | .586 | 1.706 |
|  | educational level | .004 | .019 | .008 | .191 | .848 | .803 | 1.246 |
|  | Religion | .010 | .056 | .009 | .180 | .857 | .497 | 2.012 |
|  | Ethnicity | -.049 | .038 | -.068 | -1.301 | .194 | .495 | 2.021 |
|  | age category | -.019 | .016 | -.046 | -1.164 | .245 | .847 | 1.180 |
|  | Risk perception | .162 | .048 | .127 | 3.401 | .001 | .967 | 1.034 |
| a. Dependent Variable: over all knowledge | | | | | | | | |

**For Preventive practice**

| **Hosmer and Lemeshow Test for model fitness (0.313)** | | | |
| --- | --- | --- | --- |
| Step | Chi-square | df | Sig. |
| 1 | 9.363 | 8 | **0.313** |

**Table: Multi-collinearity test of factors associated with preventive practice**

| **Coefficients^a^** | | | | | | | | |
| --- | --- | --- | --- | --- | --- | --- | --- | --- |
| Model | | Unstandardized Coefficients | | Standardized Coefficients | t | Sig. | Collinearity Statistics | |
|  |  | B | Std. Error | Beta |  |  | Tolerance | VIF |
|  | (Constant) | .020 | .091 |  | .225 | .822 |  |  |
|  | Study district | .014 | .010 | .040 | 1.396 | .163 | .945 | 1.058 |
|  | educational level | -.011 | .014 | -.023 | -.761 | .447 | .836 | 1.197 |
|  | Occupation | .078 | .061 | .037 | 1.287 | .198 | .928 | 1.078 |
|  | marital status | -.002 | .024 | -.002 | -.065 | .948 | .948 | 1.054 |
|  | religion | -.034 | .030 | -.032 | -1.127 | .260 | .956 | 1.046 |
|  | age category | -.003 | .012 | -.006 | -.216 | .829 | .878 | 1.139 |
|  | Knowledge of mode of transmission | .133 | .041 | .102 | 3.249 | .001 | .775 | 1.290 |
|  | Knowledge of Preventive measure knowledge | .553 | .033 | .553 | 16.873 | .000 | .711 | 1.406 |
|  | consequences | .105 | .040 | .096 | 2.650 | .008 | .587 | 1.703 |
|  | Knowledge of symptom | -.002 | .042 | -.002 | -.053 | .958 | .600 | 1.667 |
|  | over all knowledge | .061 | .041 | .061 | 1.491 | .136 | .453 | 2.205 |
|  | Risk perception | .040 | .037 | .031 | 1.088 | .277 | .927 | 1.079 |
| a. Dependent Variable: preventive practice | | | | | | | | |
